# Enhancement of Chemokine Function as an Immunomodulatory Strategy Employed by Human Herpesviruses

**DOI:** 10.1371/journal.ppat.1002497

**Published:** 2012-02-02

**Authors:** Abel Viejo-Borbolla, Nadia Martinez-Martín, Hendrik J. Nel, Patricia Rueda, Rocío Martín, Soledad Blanco, Fernando Arenzana-Seisdedos, Marcus Thelen, Padraic G. Fallon, Antonio Alcamí

**Affiliations:** 1 Department of Virology and Microbiology, Centro de Biología Molecular Severo Ochoa, Consejo Superior de Investigaciones Científicas – Universidad Autónoma de Madrid, Madrid, Spain; 2 School of Medicine, St James's Hospital, Trinity College Dublin, Dublin, Ireland; 3 Viral Pathogenesis Laboratory, Institute Pasteur, Paris, France; 4 INSERM U819, Paris, France; 5 Institute for Research in Biomedicine, Bellinzona, Switzerland; 6 Department of Medicine, University of Cambridge, Addenbrooke's Hospital, Cambridge, United Kingdom; University of California-Berkeley, United States of America

## Abstract

Herpes simplex virus (HSV) types 1 and 2 are highly prevalent human neurotropic pathogens that cause a variety of diseases, including lethal encephalitis. The relationship between HSV and the host immune system is one of the main determinants of the infection outcome. Chemokines play relevant roles in antiviral response and immunopathology, but the modulation of chemokine function by HSV is not well understood. We have addressed the modulation of chemokine function mediated by HSV. By using surface plasmon resonance and crosslinking assays we show that secreted glycoprotein G (SgG) from both HSV-1 and HSV-2 binds chemokines with high affinity. Chemokine binding activity was also observed in the supernatant of HSV-2 infected cells and in the plasma membrane of cells infected with HSV-1 wild type but not with a gG deficient HSV-1 mutant. Cell-binding and competition experiments indicate that the interaction takes place through the glycosaminoglycan-binding domain of the chemokine. The functional relevance of the interaction was determined both *in vitro*, by performing transwell assays, time-lapse microscopy, and signal transduction experiments; and *in vivo*, using the air pouch model of inflammation. Interestingly, and in contrast to what has been observed for previously described viral chemokine binding proteins, HSV SgGs do not inhibit chemokine function. On the contrary, HSV SgGs enhance chemotaxis both *in vitro* and *in vivo* through increasing directionality, potency and receptor signaling. This is the first report, to our knowledge, of a viral chemokine binding protein from a human pathogen that increases chemokine function and points towards a previously undescribed strategy of immune modulation mediated by viruses.

## Author Summary

Chemokines are chemotactic cytokines that direct the flux of leukocytes to the site of injury and infection, playing a relevant role in the antiviral response. An uncontrolled, unorganized chemokine response is beneath the onset and maintenance of several immunopathologies. During millions of years of evolution, viruses have developed strategies to modulate the host immune system. One of such strategies consists on the secretion of viral proteins that bind to and inhibit the function of chemokines. However, the modulation of the chemokine network mediated by the highly prevalent human pathogen herpes simplex virus (HSV) is unknown. We have addressed this issue and show that HSV-1, causing cold sores and encephalitis and HSV-2, causing urogenital tract infections, interact with chemokines. We determined that the viral protein responsible for such activity is glycoprotein G (gG). gG binds chemokines with high affinity and, in contrast to all viral chemokine binding proteins described to date that inhibit chemokine function, we found that HSV gG potentiates chemokine function *in vitro* and *in vivo*. The implications of such potentiation in HSV viral cycle, pathogenesis and chemokine function are discussed.

## Introduction

Herpes simplex virus type 1 and 2 (HSV-1 and HSV-2, respectively) and varizella zoster virus (VZV) are the three human members of the *Alphaherpesvirinae* subfamily, which establish latency in the sensory ganglia of the peripheral nervous system. Both HSV-1 and -2 are highly prevalent viruses with values around 90% for HSV-1 and 12–20% for HSV-2 in adult populations of industrialized countries, reaching up to 80% for HSV-2 in developing countries [1], [2]. Infection by HSV can be either asymptomatic, show mild symptoms in localized tissues or cause severe diseases such as stromal keratitis or herpes simplex encephalitis (HSE), with high mortality and neurologic morbidity [3]. HSV infection of neonates can result in disseminated disease including infection of the central nervous system or involve several organs with mortality reaching 80% [4]. The causes of such different outcomes following HSV infection or reactivation are unknown but involve the interplay between the virus and the immune response.

Chemokines are essential elements of the antiviral response. They constitute a family of chemotactic cytokines that orchestrate leukocyte migration to sites of injury or infection [5]. Chemokines also play relevant roles in the developing and mature nervous system [6]. The chemokine network contains more than 45 chemokines and around 20 G-protein coupled receptors (GPCR). There are 4 subfamilies of chemokines classified on C, CC, CXC and CX3C. All chemokines are secreted. CXCL16 and CX3CL1 are also present as membrane-anchored forms. The chemokine network is complex, highly regulated and promiscuous, with some receptors interacting with more than one chemokine and some chemokines binding to more than one receptor. Alterations in the chemokine network are responsible for inflammatory, autoimmune diseases and the establishment of chronic pain [7], [8]. Binding of chemokine to glycosaminoglycans (GAGs) is relevant for chemokine function. GAGs promote chemokine oligomerization, mediate retention of chemokines onto the cell surface allowing chemokine recruitment in tissues, increase their local concentration in the microenvironment surrounding the GPCR, and modulate receptor recognition [9]. Interaction of the chemokine with the GPCR triggers a signal cascade that includes stimulation of mitogen activated protein kinases (MAPKs) such as Janus-N-terminal kinase 1 and 2 (JNK1-2), extracellular signal-regulated kinase 1-2 (ERK1/2) and p38 [10]. The proper function of chemokines is essential to trigger an appropriate and effective antiviral response. An exacerbated immune response, often triggered or maintained by chemokines, may lead to immunopathology. Patients suffering from HSE present higher level of chemokine expression in the cerebrospinal fluid than healthy individuals suggesting a relevant role for chemokines in the pathogenesis of HSE [11].

Both pox- and herpesviruses express proteins that interfere with chemokine function playing relevant roles in viral cycle, immune evasion and pathogenesis [12]. One of the strategies of chemokine interference involves the expression of secreted viral proteins that bind chemokines and inhibit chemokine function [13]. These proteins have been termed viral chemokine binding proteins (vCKBP). They lack amino acid sequence similarities among themselves or with host chemokine receptors, making difficult the detection of such proteins by sequence analysis.

We, and others, have previously shown that secreted glycoprotein G (gG) from some non-human alphaherpesviruses binds to chemokines and inhibits chemokine function. Examples of such viruses are bovine herpesvirus 5 (BHV-5), equine herpesvirus 1 and 3 (EHV-1 and EHV-3) [14], [15], pseudorabies virus (PRV) [16] and infectious laryngotracheitis virus [17]. Chemokine-binding activity was not observed when supernatants of cells infected with the human viruses VZV, HSV-1 and HSV-2 were tested using different radio-iodinated chemokines [14]. In the case of VZV, the gene encoding for gG is not present within its genome. However, both HSV-1 and HSV-2 contain the open reading frame *us4* encoding gG. HSV-1 and HSV-2 gG (gG1 and gG2, respectively) are present on the viral particle and on the plasma membrane of infected cells [18]–[20]. gG2 is further processed and an N-terminal fragment is secreted to the medium of the infected cells [19], [20]. On the contrary, gG1 is not secreted, similarly to the rest of HSV glycoproteins. The functions of HSV-1 and HSV-2 gGs are not well understood. Two reports point to a role of the HSV gGs in the initial steps of entry. HSV-1 gG seems to be important for the infection of polarized epithelial cells [21]. The non-secreted portion of HSV-2 gG binds heparin and the cellular plasma membrane [22]. Deletion or disruption of *us4* attenuates HSV-1 *in vivo*, indicating that gG is a virulence factor, although the mechanism(s) beneath such phenotype are unknown [23]–[25].

The main aim of this study was to investigate the modulation of the immune system by HSV. We focused initially on identifying the function of HSV gG and its possible interaction with chemokines. We show here that secreted, soluble HSV gG (SgG) binds both CC and CXC chemokines with high affinity through the GAG-binding domain of the chemokine. Moreover, we could detect chemokine-binding activity in the plasma membrane of HSV-1 infected cells and in the supernatant of HSV-2 infected cells. Further experiments indicate that HSV-1 full-length gG and secreted, soluble HSV gG (SgG) are responsible for this activity. In complete contrast to all previously described vCKBPs, HSV-1 and HSV-2 SgGs are not inhibitors of chemokine function. Instead, they increase chemokine-mediated cell migration both *in vitro* and *in vivo* through a mechanism that involves GPCR signaling and phosphorylation of MAPKs. HSV SgGs increase the potency of the chemokine, and the directionality of cell movement. This constitutes, to our knowledge, the first description of a chemokine binding protein expressed by a human pathogen that potentiates chemokine function. The data presented here suggest the existence of a novel viral mechanism of immune modulation and provide tools to investigate the pathways controlling chemotaxis. Given the relevant roles played by chemokines in both the immune and nervous systems, enhancement of chemokine function by HSV gG may be important for HSV-mediated immunopathogenesis.

## Results

### Recombinant SgG from HSV-1 and HSV-2 binds CC and CXC chemokines with high affinity

**Figure 1:**
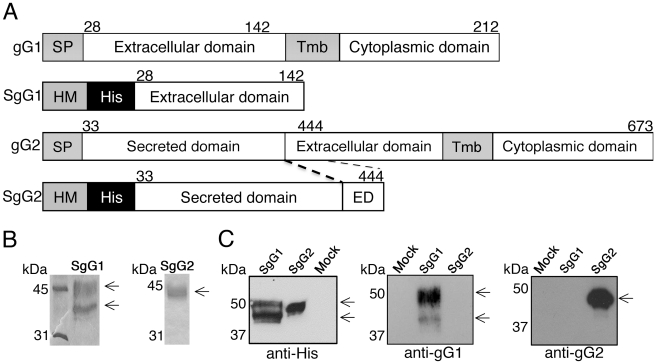
Cloning, expression and purification of HSV gG. (**A**) Schematic representation of SgG1 and SgG2 constructs used in this study. A fragment of the extracellular domain of both gG1 and gG2 was amplified and cloned into a baculovirus-expression vector. The putative signal peptide from gG was substituted by the honeybee melittin signal peptide. The position of the amino acid residues is indicated. The dashed lines indicate the fragment of the extracellular domain included in the construct. Abbreviations: SP, signal peptide; Tmb, Transmembrane domain; His, histidine tag; HM, honeybee melittin signal peptide; ED, extracellular domain. (**B**) SDS-PAGE followed by coomassie staining showing purified SgG1 (left panel) and SgG2 (right panel). (**C**) Western blots showing the detection of SgG1 and SgG2 with an anti-histidine (left panel), an anti-gG1 (middle panel) or an anti-gG2 (right panel) antibody. Molecular masses are shown in kilodaltons (kDa).

To test whether HSV gGs bind chemokines, we expressed soluble, secreted forms of gG1 and gG2 (SgG1 and SgG2, respectively), lacking the transmembrane and cytoplasmic domains, in insect cells infected with recombinant baculovirus vectors (Figure 1A; Protocol S1; Text S1). Following infection, SgG1 and SgG2 were purified from the supernatant of Hi-5 insect cell cultures by affinity chromatography and the purity of the preparation was determined by Coomassie staining (Figure 1B). We routinely obtained two separate bands when SgG1 was expressed in insect cells, probably due to different levels of SgG1 glycosylation. A monoclonal antibody raised against gG1 [18] reacted with purified SgG1 but not SgG2 (Figure 1C, middle panel) whereas a monoclonal anti-SgG2 [26] recognized SgG2 only (Figure 1C, right panel). The anti-His antibody reacted with both proteins (Figure 1C, left panel).

**Figure 2:**
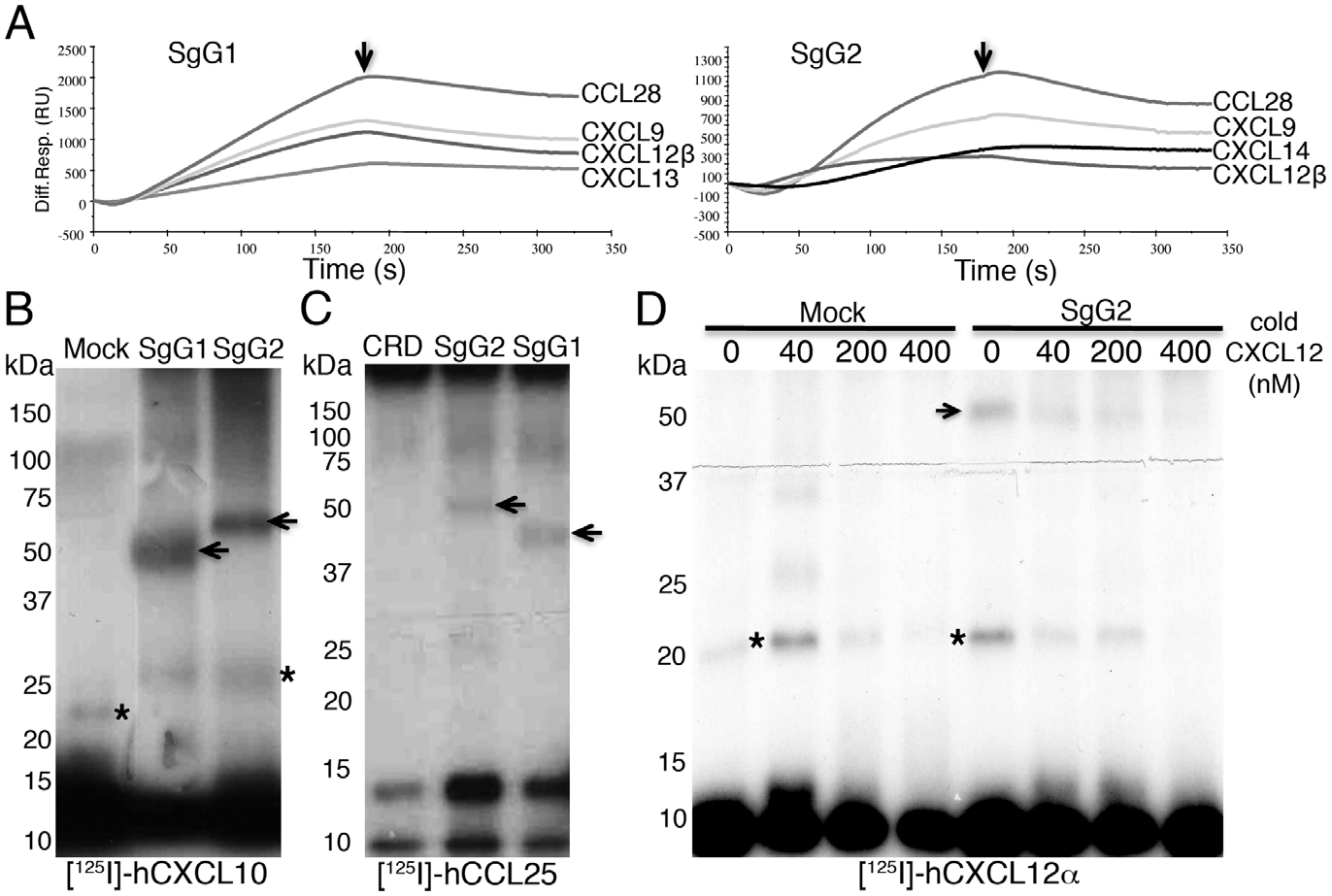
HSV-1 and HSV-2 gGs bind chemokines. (**A**) Sensorgrams depicting the interaction between chemokines and SgG1 (left) or SgG2 (right). The indicated chemokines were injected at a 100 nM concentration. The arrow indicates the end of injection. All curves were analyzed with the BiaEvaluation software and represent the interaction of the chemokine after subtraction of the blank curve. Only 4 out of 11–12 positive interactions are shown. Abbreviations: Diff. Resp., Differential response; R.U., response units; s, seconds (**B, C**) Crosslinking assays showing the interaction of HSV-SgGs with [^125^I]-hCXCL10 (**B**) and [^125^I]-hCCL25 (**C**). Recombinant purified HSV-SgGs were incubated with iodinated chemokine and crosslinked with EGS (for [^125^I]-hCCL25) or BS^3^ (for [^125^I]-hCXCL10). The samples were resolved by SDS-PAGE, fixed and visualized by autoradiography. (**D**) Crosslinking assay between [^125^I]-hCXCL12α and SgG2 in the presence of increasing concentrations of cold hCXCL12α. Molecular masses are indicated in kDa. SgG-chemokine complexes are indicated with arrows and crosslinked chemokine dimers are marked with asterisks. Abbreviations: CRD, CrmB-cysteine rich domain.

Both purified SgG1 and SgG2 were covalently coupled to BIAcore CM5 chips and tested for chemokine binding by Surface Plasmon Resonance (SPR). A screening with 44 commercially available human (h) chemokines (Protocol S2) was performed by injecting each chemokine in a BIAcore X biosensor. Both SgG1 and SgG2 bound with high affinity hCCL18, hCCL25, hCCL26, hCCL28, hCXCL9, hCXCL10, hCXCL11, hCXCL12α, hCXCL12β, hCXCL13 and hCXCL14, and SgG2 also bound hCCL22 with high affinity (Figure 2A and Table 1). As negative controls for chemokine binding we used the cysteine-rich domain (CRD) of ectromelia virus cytokine response modifier B (CrmB), previously shown to lack chemokine-binding activity [27] (not shown). The affinity constants of the interactions between SgG1, SgG2 and the different chemokines were calculated using the SPR technology (Table 1). Both SgG1 and SgG2 interacted with chemokines with high affinity, in the nanomolar range. The interaction between HSV SgGs and chemokines was also observed by cross-linking assays (Protocol S3; Text S1) using radio-iodinated recombinant hCCL25, hCXCL10, and hCXCL12α (Figure 2B–D). As a negative control we employed CrmB-CRD (Figure 2C). Competition assays with [^125^I]-hCXCL12α and increasing concentrations of cold hCXCL12α showed the specificity of SgG2-chemokine interaction (Figure 2D).

**Table 1:** Interaction affinities between SgGs from HSV-1, HSV-2 and chemokines.

Chemokine	SgG1 KD (M)	SgG2 KD (M)
hCCL18	9.02×10^−8^	2.8×10^−8^
hCCL22	n.b.	5.22×10^−9^
hCCL25	4.7×10^−9^	1.6×10^−9^
hCCL26	5.5×10^−8^	1.72×10^−9^
hCCL28	6.8×10^−8^	3.2×10^−9^
hCXCL9	3.8×10^−8^	1.23×10^−8^
hCXCL10	4.57×10^−7^	5.5×10^−9^
hCXCL11	1.09×10^−8^	6×10^−9^
hCXCL12α	3.15×10^−8^	6.5×10^−9^
hCXCL12β	7.7×10^−9^	2.2×10^−9^
hCXCL13	1.3×10^−8^	4.3×10^−9^
hCXCL14	4.2×10^−7^	4.3×10^−9^

The derived kinetic parameters and the affinity constants for the interactions between HSV SgGs and chemokines are shown. Abbreviations: n.b., not bound.

### Chemokine binding activity is present in HSV-infected cells

**Figure 3:**
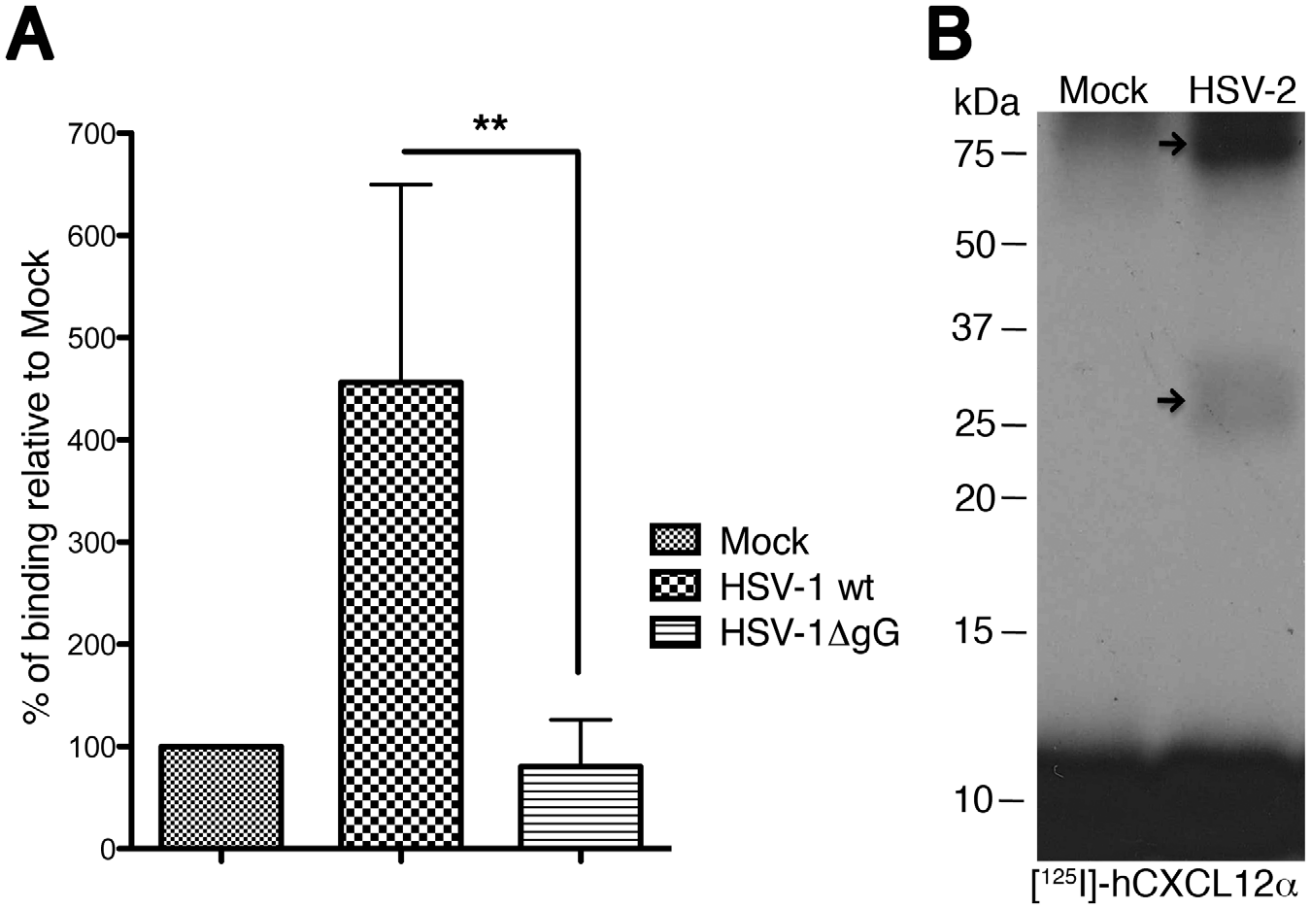
HSV-1 and HSV-2 gG expressed during infection bind chemokines. (**A**) Graph showing binding of radio-iodinated hCXCL10 to the surface of HSV-1 infected cells. Binding is observed at 14–16 h.p.i., only when cells are infected with wt HSV-1 but not when infected with a HSV-1ΔgG mutant. (**B**) Crosslinking assay showing the interaction between [^125^I]-hCXCL12α and HSV-2 gG in the supernatant of HSV-2 infected cells. The arrows point to the crosslinked complex. Abbreviations: h.p.i, hours post-infection. **P<0.01.

We addressed whether chemokine-binding activity was present in the HSV-1 infected cells. To this end we infected BHK-21 cells (Protocol S4 and S5; Text S1) with HSV-1 wt and an HSV-1 virus where expression of gG had been disrupted by the insertion of the *β-galactosidase* gene [23] and determined binding of [^125^I]-hCXCL10 to the cells 14 to 16 hours post infection (h.p.i.). We could detect chemokine binding to HSV-1 wt-infected cells (Figure 3A; Protocol S5). Binding was not observed when the deletion mutant HSV-1ΔgG was used. We also obtained supernatants from mock- or HSV-2 infected Vero cells 36 h.p.i., and performed a crosslinking assay with [^125^I]-hCXCL12α. Two bands could be detected in the crosslinking assay (Figure 3B) that could correspond to the high mannose 72 kDa precursor and the 34 kDa secreted protein produced during gG2 expression and processing [19], [20]. Another possibility is that the higher molecular weight band observed corresponds to an SgG2 dimer complexed with chemokine.

### Binding of SgG to chemokines takes place mainly through the heparin-binding domain of the chemokine

To function properly, chemokines need to interact with both GAGs and GPCRs. We investigated the chemokine domain involved in the interaction with HSV SgGs using two experimental approaches.

First, to address whether HSV SgGs could affect chemokine-receptor interaction, we performed binding assays of [^125^I]-hCXCL12α and [^125^I]-hCCL25 with MOLT-4 cells (Protocol S4 and S6) expressing endogenous hCXCR4 (the receptor for hCXCL12) and hCCR9 (the receptor for hCCL25) in the presence of SgG-containing supernatant (not shown). We also performed binding assays of [^125^I]-hCXCL12α to MonoMac-1 cells expressing endogenous hCXCR4 (not shown). As a positive control, addition of supernatant containing BHV-5 SgG inhibited [^125^I]-hCXCL12α binding to MOLT-4 cells [14] (not shown). However, similar amounts of SgG1 or SgG2 did not decrease [^125^I]-hCXCL12α binding to MOLT-4 cells, MonoMac-1 cells or [^125^I]-hCCL25 binding to MOLT-4 (not shown) compared to the mock sample. Thus, SgGs do not inhibit binding of the chemokines to their receptors.

**Figure 4:**
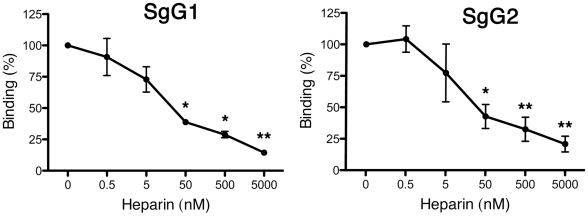
Determination of the chemokine domain involved in the interaction with HSV gGs. Heparin competition of chemokine binding to SgG1 and SgG2. hCXCL12α was injected at a concentration of 100 nM alone or in combination with the indicated increasing concentrations of heparin. The value of chemokine binding without heparin was considered 100%. All curves were analyzed with the BiaEvaluation software and represent the interaction of the chemokine after subtraction of the HBS-EP curve. The error bars represent the standard error of three independent experiments. *P<0.05; *P*<0.001.

Second, to determine the implication of the GAG-binding domain of the chemokine in the interaction with HSV SgGs we utilized the SPR technology. The amount of chemokine binding to SgGs, covalently bound to a BIAcore chip, in the absence of heparin was considered 100% of binding (Figure 4). Competition experiments showed that increasing concentrations of heparin impaired chemokine binding to both SgG1 and SgG2 in a significant manner (Figure 4). As a control, each of the different heparin concentrations used were injected independently to confirm that no direct heparin binding to the chip occurred (not shown).

In summary, these results indicate that SgG1 and SgG2 interact preferentially with the GAG-binding domain of the chemokine and do not block the binding of chemokines to cell surface specific receptors.

### Interaction of HSV SgGs with chemokines enhances chemokine-mediated cell migration

**Figure 5:**
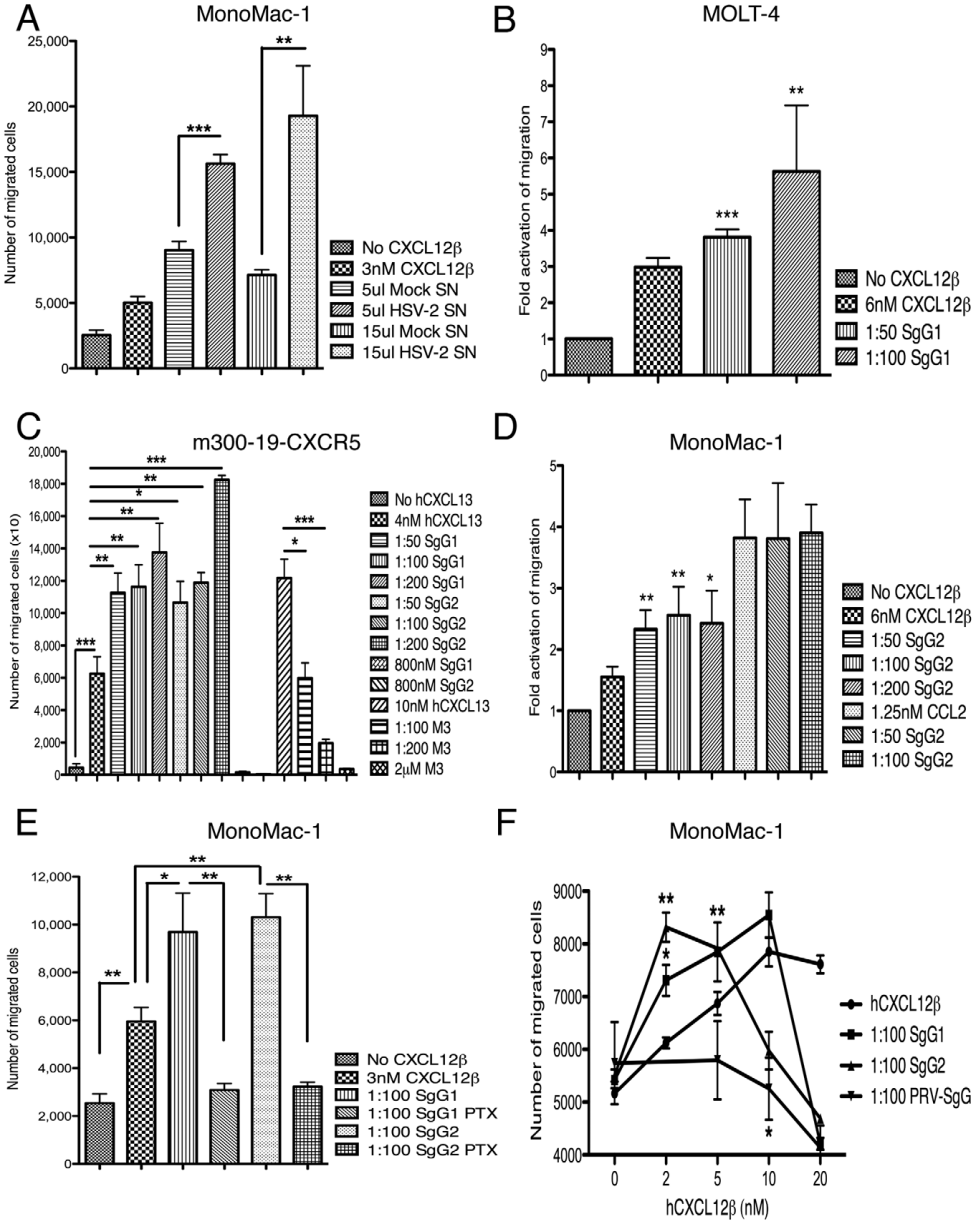
HSV SgGs enhance chemokine-mediated cell migration. MonoMac-1 cells (**A, D, E, F**), MOLT-4 (**B**), m300-19-hCXCR5 (**C**) cells were incubated with the specified chemokine in Transwell plates. The effect of mock- or HSV-2-infected supernatant (Mock SN or HSV-2 SN, respectively) (**A**), purified SgG1 (**B, C, E, F**), SgG2 (**C–F**), M3 (**C**) and PRV-SgG (**F**) was analyzed. The number of migrated cells or the fold activation of migration is depicted. (**C**) SgG1 or SgG2 require the presence of the chemokine to enhance migration since addition of either of them without chemokine did not have any effect on chemotaxis. (**D**) Binding of HSV SgGs to the chemokine is necessary for the enhancement in chemotaxis. Representation of the fold activation of migration observed when cells were incubated with either hCXCL12β or hCCL2 in the absence or presence of increasing concentrations of HSV-2 gGs. (**E**) Addition of pertussis toxin (PTX) inhibits SgG-mediated enhancement of chemotaxis. Graph showing the effect of PTX addition on HSV SgGs enhancement of hCXCL12β-mediated chemotaxis. The number of migrated MonoMac-1 cells is represented. (**F**) HSV SgGs displace the hCXCL12β chemotactic curve towards lower concentrations of chemokine. MonoMac-1 cells were incubated with increasing concentrations of hCXCL12β in the absence or presence of a 1∶100 molar ratio of HSV SgGs or PRV-SgG. (**A–F**) Error bars indicate standard deviation values obtained from triplicate samples (A, C, E, F). One representative experiment of at least three is shown. In B and D, error bars represent the standard deviation in the fold activation obtained using three independent experiments performed in duplicate. *P<0.05; **P<0.01; ***P<0.001.

We, and others, have previously shown that gG encoded by several non-human alphaherpesviruses inhibits chemotaxis [14]–[17], [28]. To examine the functional role of the interaction between HSV SgGs and chemokines we performed cell migration experiments. First we addressed whether the chemokine-binding activity observed in the supernatant of HSV-2 infected cells could have any effect on chemotaxis. We incubated CXCL12β with supernatant from mock- or HSV-2-infected cells and performed a chemotactic assay with MonoMac-1 cells (monocyte-like), a cell line that expresses hCXCR4, the receptor for hCXCL12. The supernatant from HSV-2-infected cells significantly enhanced chemokine function in a dose dependent manner when compared to the supernatant from mock-infected cells (Figure 5A). To address whether this effect could be due to SgG, we performed chemotactic experiments using several cell lines and recombinant protein. Incubation of SgG1 with hCXCL12β resulted in higher MOLT-4 migration (Figure 5B). A similar result was obtained with SgG2 whereas BHV-5 SgG inhibited hCXCL12β migration (not shown). We then incubated SgG1 and SgG2 with hCXCL13 and tested their effect on mouse B cells (m300-19) stably transfected with hCXCR5, the receptor for hCXCL13 (Figure 5C, Protocol S4). Inhibition of migration was observed with the vCKBP M3, as expected [29], [30] (Figure 5C). However, SgG1 and SgG2 required the presence of the chemokine and were not able to induce chemotaxis on their own (Figure 5C). The parental m300-19 cells, which do not express hCXCR5, did not respond to the hCXCL13 stimulus (not shown). To test whether binding to the chemokine was necessary for the enhancing effect, we performed chemotaxis experiments using MonoMac-1, a cell line expressing hCXCR4 and hCCR2, the receptor for hCCL2, a chemokine not bound by HSV SgGs (Figure 2 and Table 1). The enhancement in chemotaxis mediated by SgGs required SgG-chemokine interaction since SgG2 did not have any effect on the chemotactic properties of hCCL2 (Figure 5D), whereas it was able to potentiate hCXCL12β. A similar result was obtained with SgG1 (not shown). In all cases, the enhancement in chemotaxis was dose dependent and significant.

The effect of SgGs on chemotaxis was dependent on G protein activation since addition of pertussis toxin (PTX) inhibited both hCXCL12β-mediated cell migration and its enhancement mediated by SgGs (Figure 5E). Finally, we examined the effect of SgG1 and SgG2 on hCXCL12β-mediated cell migration utilizing increasing concentrations of hCXCL12β and a constant molar ratio (1∶100) between the chemokine and SgG (Figure 5F). The effect of hCXCL12β on *in vitro* cell migration had the characteristic bell-shaped curve (not shown). As a control we used PRV-SgG, which inhibited chemokine-mediated migration [16]. However, both SgG1 and SgG2 enhanced the potency of hCXCL12, displacing the chemotactic bell-shaped curve towards lower concentrations of the chemokine.

### HSV SgG enhances chemokine efficiency and directionality

**Figure 6:**
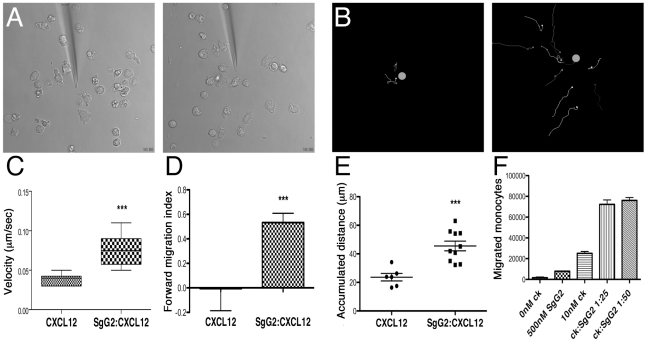
Analysis of SgG2 induced enhancement of chemotaxis by time-lapse video microscopy. (**A**) Selected frames from Videos S1 and S2 showing migration towards CXCL12 (left) or CXCL12-SgG2 (right). (**B**) Migrating cells were tracked and their progressive trajectories were plotted according to the recorded xy coordinates. Orange dots indicate the real position of the micropipette dispensing CXCL12 (left) or CXCL12-SgG2 (right). Each line represents the path followed by one cell during 10 min at the initial phase of chemotaxis. (**C**) The velocity of cell movement, (**D**) forward migration index and (**E**) total traveled distance by cells migrating towards the micropipette dispensing CXCL12 or CXCL12-SgG2 were plotted. Representative data from 6 cells (CXCL12) and 10 cells (CXCL12-SgG2) migrating at the initial time period are shown. Time-lapse videos were analyzed using Image J 1.43 software. The trajectories of the tracks, velocities, FMI and distances traveled were calculated using Manual Tracking and Chemotaxis Tool version 1.01 plugging. The analysis of 1 representative video out of three is shown. (**F**) Representation of migrated monocytes in the presence of CXCL12 alone or in combination with SgG2 using the transwell technology. 1 representative experiment out of three is shown. Error bars indicate standard error values. *:*P*<0.05; **:*P*<0.01; ***:*P*<0.001.

To analyze the impact of HSV SgG on different aspects of chemotaxis in real time we performed time-lapse video microscopy using freshly isolated human monocytes and hCXCL12β. The chemokine, alone or in combination with SgG2, was released from a micropipette with constant backpressure. Analysis of tracks recorded by time-lapse video microscopy from cell cultures stimulated with CXCL12β (Video S1) or CXCL12β-SgG2 (Video S2) clearly showed that chemotaxis in the presence of the viral protein was enhanced, compared to the migration towards the chemokine alone (Videos S1, S2 and Figure 6B). SgG2 was not able to trigger migration in the absence of the chemokine (Video S3). Consistent with our data from transwell assays (Figure 5), SgG2 greatly enhanced the number of human monocytes that moved towards a given concentration of the chemoattractant (Figure 6). The cells sensed the chemokine gradient from longer distance to the dispensing pipette than when chemokine was dispensed alone. Chemotactic parameters, i.e. velocity, FMI and distance traveled, were calculated during an initial 10-min period. The velocity of the cell movement and the Forward Migration Index (FMI), i.e. the ratio of the net distance the cell progressed in the forward direction to the total distance the cell traveled, were significantly increased when SgG2 was bound to CXCL12β (Figure 6C, D). Moreover, the cells travelled a longer distance when the chemokine and SgG2 were dispensed together than when the chemokine was dispensed alone (Figure 6E). Similar results were obtained when using CXCL12α (not shown). Transwell experiments performed in parallel with freshly isolated human monocytes confirmed the SgG2-mediated enhancement of CXCL12β chemotaxis observed by video microscopy (Figure 6F).

### Interaction of HSV SgGs with chemokine increases chemokine-mediated signaling

**Figure 7:**
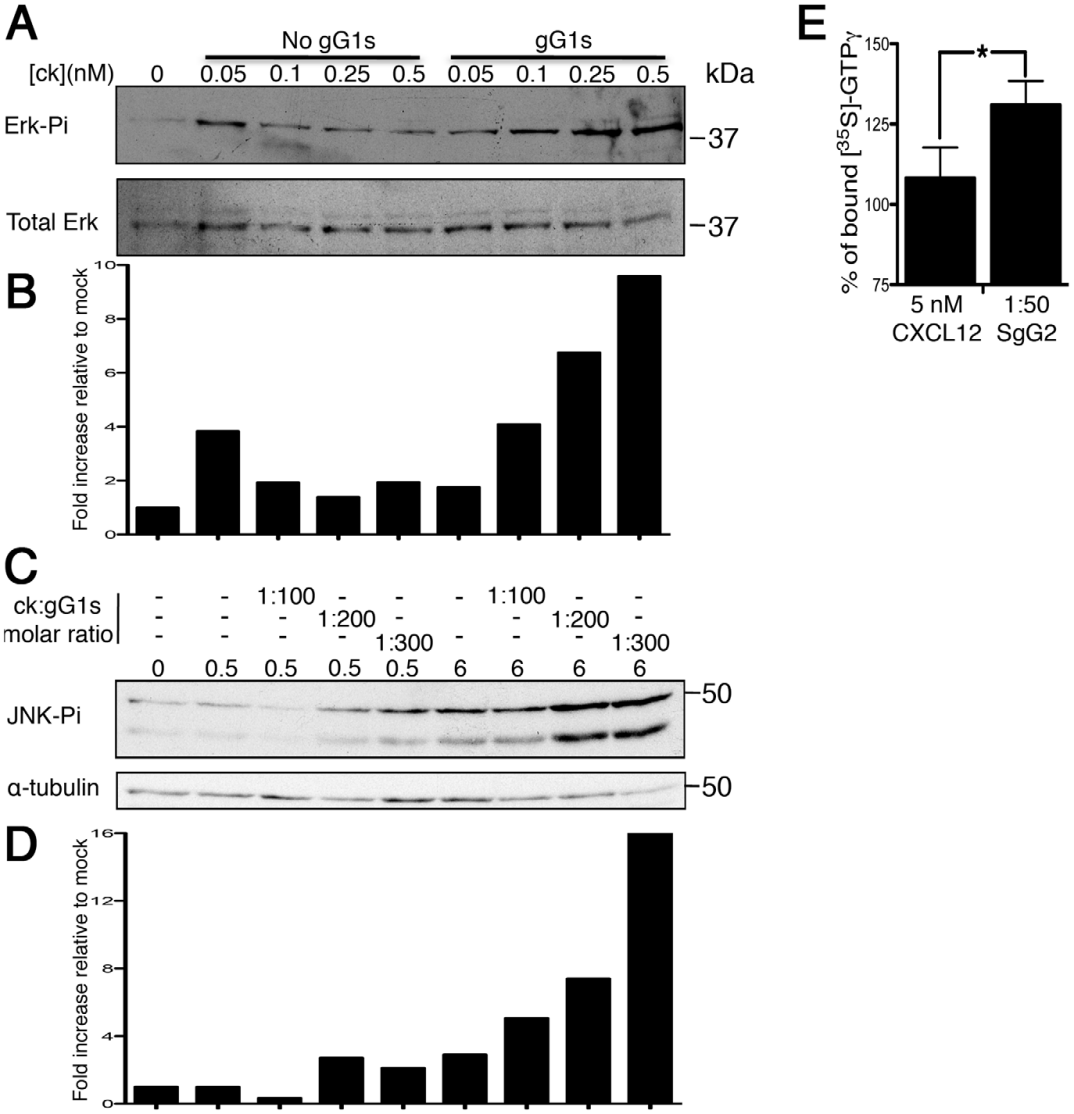
HSV SgG enhances chemokine-mediated signaling. (**A**) Western blots showing activation of ERK (p-ERK, top blot) and loading control (Total ERK, bottom blot) in MonoMac-1 cells incubated with CXCL12 alone or with a constant 1∶200 molar ratio of chemokine∶SgG1. (**C**) Western blot showing the effect of increasing concentrations of HSV SgG1 on chemokine-mediated JNK phosphorylation (top blot). As loading control, the blots were stripped and incubated with anti-alpha-tubulin (bottom blot). (**B and D**) Graphs depicting the results obtained after performing a densitometer analysis of the blots. The densities obtained from each of the lanes in the MAPKs blots were normalized to the loading controls and later to the mock sample. (**E**) Graph showing the percentage of [^35^S]-GTPγ binding to CXCR4 mediated by CXCL12 alone or with SgG2 (considering no CXCL12 as 100%). The results of combining three independent experiments performed in duplicate are shown. Error bars indicate standard deviation values. * *P*<0.05.

MAPKs are involved in several cellular processes including cell migration [31]. Binding of chemokine to its receptor activates a signaling cascade that involves phosphorylation and, thereby, activation of MAPKs. Incubation of MonoMac-1 cells with low doses of hCXCL12β resulted in low activation of MAPKs (Figure 7). Pre-incubation of different concentrations of hCXCL12β with a constant molar ratio (1∶200) of SgG1 enhanced the phosphorylation of ERK (Figure 7A and B). The SgG1-mediated increase in the phosphorylation of JNK1-2 was dose-dependent (Figure 7C and D). Similar results were obtained with SgG2 (not shown). Densitometer analysis of the blots shows a dose-dependent enhancement of MAPK activation in the range of 5 fold for both ERK and JNK at the highest chemokine concentration. These results showed, using a different biological assay, a similar enhancement of chemokine activity mediated by HSV SgGs. Activation of CXCR4 results in the dissociation of GDP from the Gαβγ heterotrimer followed by association of GTP to the Gα subunit. In order to measure the effect of HSV SgG on receptor occupancy we performed a [^35^S]-GTPγS binding assay. The results show that the incubation of CXCL12β with SgG results in higher levels of [^35^S]-GTPγS incorporation (Figure 7E).

### HSV-2 SgG increases chemotaxis *in vivo*

**Figure 8:**
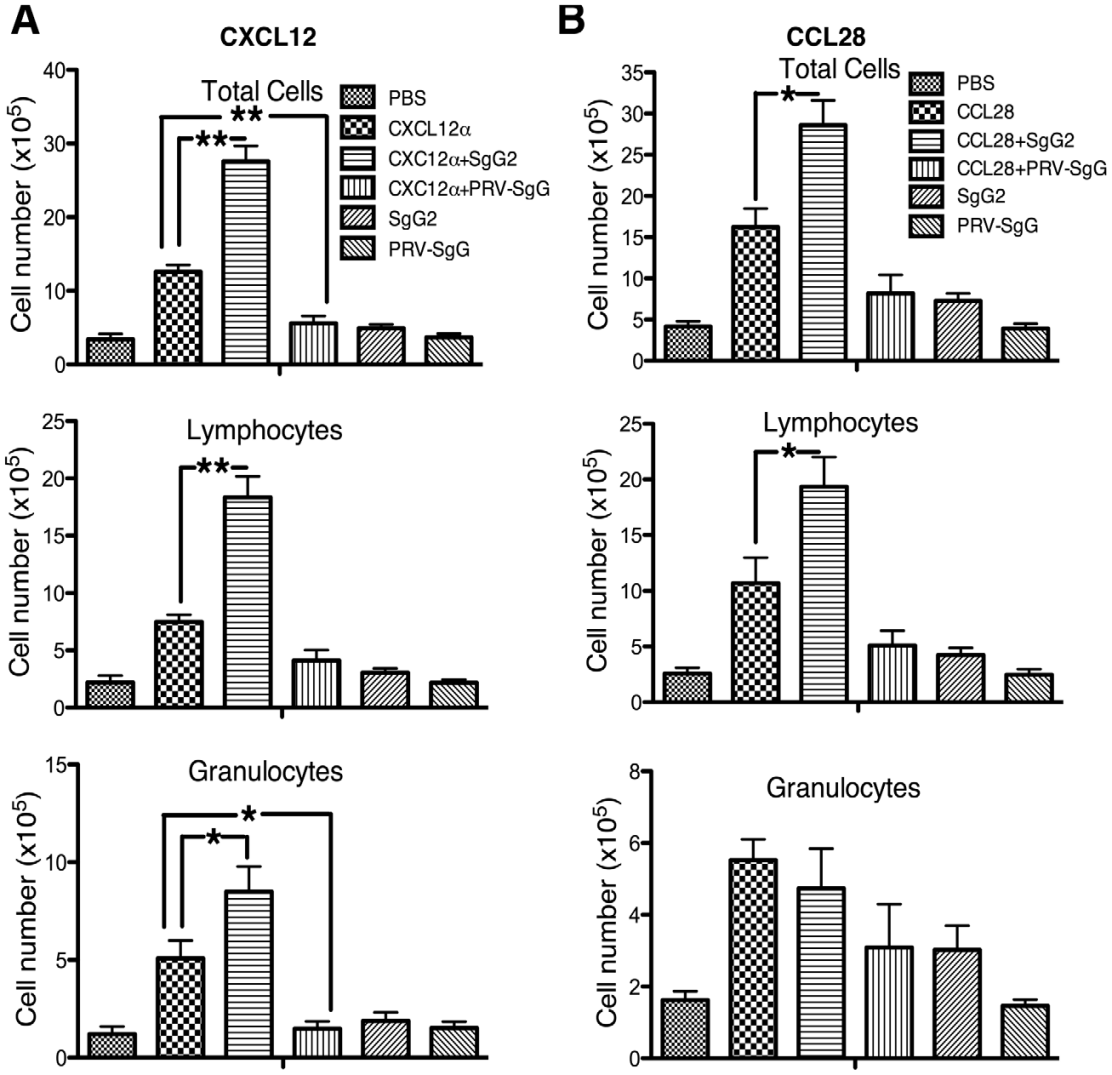
HSV-2 SgG enhances chemokine-mediated cell migration *in vivo*. CXCL12α (**A**) or CCL28 (**B**) were injected into dorsal air pouches in mice alone or in combination with HSV-2 SgG or PRV SgG. Cell migration into the air cavity was monitored. Cells were extracted and identified by flow cytometry with specific markers. The number of total leukocytes (top), lymphocytes (middle) and granulocyte cells (bottom graph) is represented. Data are mean and SEM from 5–6 mice per group and are representative of 2–3 separate experiments. *:*P*<0.05; **:*P*<0.001.

We tested the functional relevance of SgG2-chemokine interaction *in vivo* using the mouse air pouch model, by performing injections of chemokine alone or in combination with SgG2. Injection of 0.2 µg of mCXCL12α or mCCL28 induced the migration of leukocytes into the air cavity (Figure 8). The presence of 2 µg SgG2 enhanced CXCL12α-mediated migration (Figure 8A) of total leukocytes (top panel, *P*<0.001), lymphocytes (middle panel, *P*<0.001) and granulocytes (bottom panel, *P*<0.05). As a control, we used 2 µg recombinant secreted gG from PRV (PRV-SgG), a vCKBP shown to inhibit chemotaxis [16]. PRV-SgG significantly inhibited CXCL12α-mediated chemotaxis of total leukocytes (top panel, *P*<0.001) and granulocytes (bottom panel, *P*<0.05). CCL28-mediated chemotaxis (Figure 8B) of total leukocytes (top panel) and lymphocytes (middle panel) was significantly increased by SgG2 (*P*<0.05), whereas the migration of granulocytes (bottom panel) was not affected by SgG2. This could be explained by the specificity of CCL28 in driving T cell chemotaxis. In contrast to the inhibition observed when CXCL12 was used, PRV-SgG did not significantly inhibit CCL28-mediated chemotaxis. This may be due to uncontrolled factors such as the stability of the PRV-SgG-CCL28 complex in vivo or the indirect activation of other chemoattractants that may also induce migration. Injection of SgG2 or PRV-SgG alone, in the absence of chemokine, did not result in differences in leukocyte chemotaxis when compared to PBS injection.

## Discussion

HSV glycoproteins play relevant roles in the viral cycle and pathogenesis, and constitute promising vaccine candidates [32], [33]. Among all HSV glycoproteins, gG is the least well characterized and its function has not been fully elucidated. A role for HSV gG on virus entry has been suggested. HSV-1 gG seems to be important for the infection of, but not initial binding to, polarized cells through the apical surface [21]. The non-secreted domain of HSV-2 gG could participate in initial interaction of the virion with the cell surface [21], [22]. A synthetic peptide encompassing amino acids 190–205 from the secreted domain of HSV-2 gG was found to have a proinflammatory role *in vitro* when bound to the formyl peptide receptor [34]. However, until present, no function has been attributed to the full-length secreted portion of HSV-2 gG. Here, we have investigated the function of secreted forms of gG from HSV-1 and HSV-2. We show for the first time a chemokine-binding activity both in HSV-1 infected cells and in the supernatant of HSV-2 infected cells. Disruption of the HSV-1 gG expression abrogated chemokine binding suggesting that HSV gG is the protein responsible for the interaction. We could indeed show that both HSV-1 and HSV-2 SgG bind with high affinity, in the nanomolar range, CC and CXC chemokines. This interaction was demonstrated by the use of two different experimental approaches: crosslinking assays and SPR. Finally, and more importantly, we describe the first vCKBP, to our knowledge, with the ability to increase chemotaxis both *in vitro* and *in vivo* by enhancing the potency of the chemokine and the directionality of cell migration. HSV SgGs enhancement of chemotaxis required the interaction with the chemokine through the chemokine GAG-binding domain and involved signaling through the GPCR and activation of MAPKs. We confirmed that supernatant containing gG secreted following HSV-2 infection enhances chemokine-mediated migration of leukocytes. Moreover, in preliminary experiments we have found that membrane-anchored gG expressed during HSV-1 replication in cell culture also enhances chemokine activity (N.M.-M. and A.V.-B., unpublished data).

During evolution, viruses have developed strategies to modulate the host immune response. Inhibition of chemokine function through the expression of vCKBP is a common strategy in members of the *Poxviridae* family [12], [35] indicating the importance of chemokines in antiviral defense. In the *Herpesviridae* family, however, there are only three examples of vCKBP reported to date, two of them expressed by animal viruses -gG from alphaherpesviruses and M3 from murine herpesvirus 68- and one expressed by a human pathogen, pUL21.5 encoded by human cytomegalovirus [14], [36]. In addition, interaction of HSV gB with a reduced number of chemokines has been reported [37]. However, this interaction was of low affinity, in the micromolar range [37] compared to the nanomolar range observed for all vCKBP [14], [16], [17], [29], [30], [36]. Moreover, gB did not seem to have an effect on chemotaxis [37]. Nearly all previously described vCKBP have been shown to inhibit chemotaxis either *in vitro* or *in vivo*. As a general rule, vCKBPs inhibit chemokine function through impairing chemokine-receptor interaction or chemokine presentation by GAGs [38]. For instance, gG from some animal alphaherpesviruses blocks chemokine interaction with its receptor [14], [28] and with GAGs [14] inhibiting chemotaxis [14], [16], [17]. To date, there are no reports of a vCKBP that potentiates chemokine function either *in vitro* or *in vivo*. HSV SgG is, therefore, the first vCKBP described, to our knowledge, which enhances chemokine function both *in vitro* and *in vivo*.

Our studies with SgG1 and SgG2 show that these viral proteins interact with the GAG-binding domain of the chemokines and enhance the chemokine activation of GPCRs. Chemokine-GAG interaction is required for correct chemokine function *in vivo* [9]. Several reports show that GAG-binding deficient chemokines are functionally impaired *in vivo* and when *in vitro* migration and invasion assays are performed [39], [40]. GAGs also modify chemokine quaternary structure and this seems to be required for chemokine function [39], [41]. We propose a model in which SgG1 and SgG2 act similarly to the GAGs, maybe by increasing the local chemokine concentration, modifying the chemokine quaternary structure or improving chemokine presentation to the receptor so that signaling is enhanced. This would cause the observed activation of chemokine signaling at lower doses of chemokine when gG is present. This contrasts with the related gGs encoded by non-human herpesviruses, which have been shown to inhibit chemokine-mediated signal transduction and cell migration [14]–[17]. It appears that HSV-1 and HSV-2 have evolved a vCKBP to enhance, rather than to inhibit, chemokine function, and this may represent an advantage to these human herpesviruses.

The functional relevance of chemokine enhancement in HSV life cycle and pathogenesis is unknown. The role of alphaherpesvirus gG *in vivo* is not fully understood. Results presented in several reports indicate that gG from animal alphaherpesviruses is relevant for pathogenesis and immune modulation [15], [17]. There are currently no data on the role of HSV-2 gG on pathogenesis. Three independent reports show that lack of gG expression in HSV-1 leads to different degrees of virus attenuation [23]–[25]. Thus, lower viral titers were detected in mouse tissues infected through scarification of the ear with an HSV-1 mutant lacking gG [23]. A double *us3*/*us4* deletion mutant (with *us3* encoding a kinase and *us4* encoding gG) was attenuated following intracranial injection [24]. However, the relative contribution of either protein in that animal model could not be defined. Mutation of the *us4* gene by the use of transposon Tn5 resulted in a HSV-1 mutant that was less pathogenic, was deficient in its ability to replicate in the mouse central nervous system and caused a delay in encephalitis induction [25]. The mechanisms of attenuation of HSV-1 gG mutant viruses are unknown, but the discovery that HSV-1 gG enhances chemokine function points to a role of HSV gG on deregulation of chemokine function that could explain the lower pathogenicity observed with the mutant viruses.

Although there are not yet systematic analyses on the expression of all known chemokines on the tissues relevant for HSV infection, the information obtained by several laboratories supports the relevance of chemokines on HSV infection and pathogenesis. The expression of some chemokines is upregulated upon HSV-1 and HSV-2 infection [42], [43] leading to leukocyte infiltration, which may be as pathogenic as viral infection [44]. In fact, chemokines are important in HSE pathogenesis in humans [11]. Deficiency in CXCR3 or CCR5 increases susceptibility to genital HSV-2 infection although through different mechanisms [43], [45]. Interestingly, the lack of CXCR3 does not result in lower leukocyte recruitment. On the contrary, CXCR3^−/−^ mice show an increase in viral titers, infiltrating cells and neuropathology accompanied by a higher level of cytokine and chemokine expression in brain and spinal cord [46]. Differences were observed between CXCL10^−/−^ and CXCR3^−/−^ (the receptor for CXCL10) mice when challenged with ocular HSV-1 infection [47], [48]. However, CXCR3^−/−^ responded like CXCL9^−/−^ or CXCL10^−/−^ in a genital model of HSV-2 infection [46]. There are also differences in susceptibility depending on the route of infection and the nature of the pathogen employed. The redundancy of the chemokine network may be beneath some of these differences and discrepancies.

The chemokines bound by SgG1 and SgG2 are expressed in tissues relevant for HSV infection, replication and spread. Among other cell types, mucosal epithelial cells express CCL25, CCL28 and CXCL13: (1) CCL25 expression is upregulated during oral wound healing [49]; (2) CCL28 is expressed in airway epithelial cells [50]; and (3) CXCL13 is required for the organization and function of the nasal-associated lymphoid tissue [51]. Human corneal keratinocytes express CXCL9, CXCL10 and CXCL11, expression that can be further induced by proinflammatory cytokines [52]. CXCL14 expression in taste-bud cells is remarkably high and secreted to the saliva [53]. Among other tissues, CXCL12 is expressed in nervous tissues where it has been suggested to play a role in leukocyte extravasation [54]. CXCL12 also induces migration of neural progenitors, is required for axonal elongation and pathfinding, is relevant for neurotoxicity and neurotransmission in the adult nervous system and contributes to chronic pain [6], [8]. Thus, modulation of the activity of chemokines mediated by gG1 and gG2 could occur in tissues infected by HSV and play a role in HSV biology.

Enhancement of chemokine function by HSV SgGs could impact at least four different scenarios relevant for HSV spread and pathogenesis. First, enhancement of GPCR signaling could aid in the early steps of infection and in viral replication. In fact, MAPK activation is required for efficient HSV replication [55]. In this scenario gG1, due to its presence in the viral particle and at the plasma membrane of the infected cells, may play a more relevant role than gG2, which is processed secreting its chemokine-binding domain. Second, increase in the level of infiltrating leukocytes, or differences in the composition of such infiltrate, could skew the immune response and favor viral replication. The fact that HSV SgGs only bind 11–12 out of 45 human chemokines with high affinity suggests the existence of a selectivity and specificity in the modulation of the immune response. Third, enhancement in the migration of a particular leukocyte population could recruit cells that may be subsequently infected by HSV, enhancing viral load. Fourth, modulation of chemokines present in the nervous system, such as CXCL12, could play a role in the initial infection of the ganglia, sites of HSV latency, and increase the ability of HSV to persist and cause disease. The impact of HSV gG-chemokine interaction on HSV biology requires further characterization.

In summary, this is the first report of a vCKBP that enhances chemokine function and suggests a novel mechanism of immune modulation mediated by a highly relevant and prevalent human pathogen. The findings reported here shall foster further investigations on the role of HSV gG on pathogenesis and immune modulation and will allow the design of novel immunomodulators, antiviral drugs and tools to study chemokine function.

## Materials and Methods

### Ethics statement

All animal experiments were performed in compliance with Irish Department of Health and Children regulations and approved by the Trinity College Dublin's BioResources ethical review board. Human peripheral blood monocytes were prepared from buffy coats obtained from the local donor bank (“Servizio Trasfusione, Svizzera Italiana”, CH-6900 Lugano, Switzerland), with oral consent from the donors according to Swiss regulations. The use of buffy coats was approved by the institutional review board “Comitato Etico Cantonale, CH-6501 Bellinzona, Switzerland” and the experimental studies were approved by the “Dipartimento della Sanitá e della Socialitá”.

### Determination of SgG-chemokine binding specificity and affinities using SPR technology

The interactions between chemokines and SgGs and their affinity constants were determined by SPR technology using a Biacore X biosensor (GE Healthcare) as previously described [16]. Both proteins were dialyzed against acetate buffer (pH 5.0 for SgG1 and pH 5.5 for SgG2) prior to amine-coupling of the recombinant proteins in CM5 chips. Chemokines that did not bind under kinetic conditions were considered negative and not taken into further consideration for the study. In competition experiments with heparin the chemokine was injected at 100 nM alone or with increasing concentrations of heparin in HBS-EP buffer (10 mM Hepes, 150 mM, NaCl, 3 mM EDTA, 0.005% (vol/vol) surfactant P20, pH 7.4) at a flow rate of 10 µl/min, and association and dissociation were monitored. All Biacore sensorgrams were analyzed with the software Biaevaluation 3.2. Bulk refractive index changes were removed by subtracting the reference flow cell responses, and the average response of a blank injection was subtracted from all analyte sensorgrams to remove systematic artifacts.

### Competition of chemokine binding to cells

Competition experiments were carried out incubating 0.5 pmol of [^125^I]-hCCL25 or [^125^I]-hCXCL12α with or without different concentrations of SgGs (or baculovirus supernatants) at 4°C in binding medium (RPMI 1640 containing 1%FBS and 20 mM HEPES pH 7.4) during 1 h at 4°C. Then, 3×10^6^ MOLT-4 or MonoMac cells were added to the mixture and incubated for further 2 h at 4°C with gentle agitation, subjected to phthalate oil centrifugation, washed twice with PBS, and cell-bound chemokine was determined using a gamma-counter.

### Chemotaxis assays

Chemokines were placed in the lower compartment of 24-well transwell plates (Costar) or in 96-well ChemoTx System plates (Neuro Probe Inc., MD, USA) with or without recombinant gGs in RPMI 1640 containing 1% FBS. MOLT-4, MonoMac-1, m300-19 and m300-19-hCXCR5 cells were placed on the upper compartment (3×10^5^ cells in the 24-well transwell plate and 1.25×10^5^ cells in the 96-well ChemoTx System plate, with the exception of m300-19-hCXCR5 where 2.5×10^5^ cells were used). To test the effect of supernatant from mock- or HSV-2-infected cells in chemotaxis, the cells were infected in the presence of Optimem (Gibco) and the supernatants were collected 36 h.p.i. These supernatants were inactivated with psoralen as previously described [56] and concentrated 10 times using a Vivaspin 500 (Sartorius) prior to use. Both chambers were separated by a 3 µm (for MOLT-4, MonoMac-1 cells and monocytes) or 5 µm (for m300-19 and m300-19-hCXCR5 cells) pore size filter. The plates were incubated at 37°C during 2–4 h and the number of cells in the lower chamber was determined using a flowcytometer (for 24-well transwell plates) or by staining the cells with 5 µl of CellTiter 96 aqueous one solution cell proliferation assay (Promega, WI, USA) during 2 h at 37°C and measuring absorbance at 492 nm, with the exception of monocytes and m300-19-hCXCR5 which were counted with a light microscopy. When the CellTiter 96 aqueous one solution cell proliferation assay was used, known amounts of cells were incubated with the CellTiter solution to quantify the number of migrated cells. When used, PTX was incubated with MonoMac-1 cells overnight at a concentration of 0.1 µg/ml, prior to the chemotaxis experiment.

### Isolation of human monocytes from blood and time-lapse video microscopy

Monocytes were isolated from blood of healthy donors by negative selection using Monocyte Isolation kit II (MACS Miltenyi Biotec). Peripheral blood mononuclear cells (PBMBs) were isolated from heparinized blood by Ficoll (Lymphoprep) gradient centrifugation. Cells were resuspended in MACs buffer and incubated with FcR blocking reagent at 4°C. Monocytes were purified by negative selection according to the manufacturer's protocol. Time-lapse video microscopy analysis of chemotaxis was performed immediately with a Leica DI6000 microscope stand connected to a SP5 scan head equipped with a temperature controlled chamber (Cube, LIS, Basel). Freshly isolated monocytes were placed in a humidified and CO_2_-controlled incubator, which was mounted on the microscope stage (Brick, LIS, Basel). Cells were resuspended in D-PBS containing calcium and magnesium (Invitrogen) supplemented with 1% FBS, Pen/Strep, 0.04 mM sodium pyruvate, 1 mg/ml fatty acid free BSA (Sigma), 1 mg/ml glucose (Fluka). Cells were plated on glass bottom petri-dishes (MatTek cultureware) which were coated previously with D-poly-lysine (5 µg/ml) and subsequently overlaid with 3 µg/ml VCAM-1 (BD Biosciences) at 4°C overnight. Before plating the cells, coated-dishes were treated with PBS containing FBS and BSA to block non-specific binding. Chemokine was dispensed with a micropipette (Femtotip II, Eppendorf) controlled by a micromanipulator (Eppendorf) at a constant backpressure of 30 hPa (Femtojet, Eppendorf).

### Activation of mitogen activated protein kinases

Chemokine alone or in combination with SgGs was added to 10^6^ MonoMac-1 cells and incubated during 1 min at 37°C. Cells were lysed in lysis buffer (20 mM triethanolamine pH 8.0, 300 mM NaCl, 2 mM EDTA, 20% glycerol, 1% digitonin and proteinase inhibitors). The lysate was analyzed by western blotting using anti-phospho-ERK, anti-phospho-P38 (Cell Signaling Technology) or anti-phospho-JNK1/2 polyclonal antibodies (Abcam). Blots were scanned and the densities of the bands were analyzed and compared with the Image J 1.43 software normalizing the densities obtained from each band from the MAPK blots to their respective loading controls.

### Air pouch model

Age-matched female C57BL/6 mice from Harlan (Bicester, U.K.) were housed in a specific pathogen-free facility in individually ventilated and filtered cages under positive pressure. All animal experiments were performed in compliance with Irish Department of Health and Children regulations and approved by the Trinity College Dublin's BioResources ethical review board.

Dorsal air pouches were induced in mice as described [57]. In brief, 5 ml of sterile-filtered air was injected subcutaneously into the dorsal skin of mice, with air pouches re-inflated with 3 ml of sterile air 3 days later. The dorsal air pouches of groups of 5–6 mice were injected 2 days later with 0.2 µg chemokine alone or in combination with 2 µg SgG. Mice were killed and air pouches were lavaged with PBS 3 h later. The air pouch aspirate was centrifuged and total leukocytes cells were counted.

Cells were stained with a panel of mAbs for surface markers for flow cytometric cell characterization as described [58]. mAbs used were from BD Biosciences; PerCP anti-CD4 (RM4-5), PerCP-Cy5.5 anti-CD19 (1D3), PerCP anti-CD8a (53-6.7), PerCP anti-CD11b (M1/70) and eBioscience: PE anti-Ly6G (RB6/8C5). Cells were defined as lymphocytes (CD4+CD8+CD19+) and Ly6G^hi^CD11b^+^ granulocytes (neutrophils). Data were collected on a CyAn (Beckman Coulter) and analyzed using FlowJo (Tree Star). Quadrants were drawn using appropriate isotype-controls and data plotted on logarithmic scale density- or dot-plots.

### Statistical analysis

Statistical analyses of data were performed with the program GraphPad Prism. The significant value (*P* value) for the parameters measured in all assays was calculated using the student's t-test with the exception of the ones obtained in the air-pouch model experiments which was calculated using the one-way analysis of variance (ANOVA).

## Supporting Information

Protocol S1 **Generation of recombinant baculoviruses and purification of recombinant proteins.** Description of the procedure employed to generate recombinant baculoviruses and purify recombinant proteins.
(DOC)

Protocol S2 **Recombinant chemokines.** Relation of recombinant chemokines used in this report.
(DOC)

Protocol S3 **Cross-linking experiments.** Explanation of the method used to perform cross-linking.
(DOC)

Protocol S4 **Cells and viruses.** Cells and viruses utilized in this report.
(DOC)

Protocol S5 **Chemokine binding to infected cells.** Description of the method employed to analyze binding of radiolabeled chemokine to infected cells.
(DOC)

Protocol S6 **Competition of chemokine binding to cells.** Explanation of the procedure performed to determine the effect of HSV SgG on radiolabeled chemokine binding to cells.
(DOC)

Text S1 Includes references used in Protocol S1, Protocol S3, Protocol S4.
(DOC)

Video S1 **Migration of human monocytes towards CXCL12β.** Freshly isolated monocytes from blood of healthy donors were plated on glass bottom cover slips coated with Poly-D-lysine and VCAM-1. CXCL12β (100 nM) was dispensed from a micropipette with constant backpressure. Time-lapse video was recorded at 10 seconds interval with DIC optics at 63× magnification. 1 representative video of three is shown. DiVX software should be used to open and play this video.
(AVI)

Video S2 **Migration of human monocytes towards CXCL12β-SgG2.** Freshly isolated monocytes from blood of healthy donors were plated on glass bottom cover slips coated with Poly-D-lysine and VCAM-1. CXCL12β (100 nM) was pre-incubated with SgG2 in a molar ratio 1∶50 during 30 min at RT. CXCL12 and SgG2 complex was dispensed from a micropipette with constant backpressure. Time-lapse video was recorded at 10 seconds interval with DIC optics at 63× magnification. 1 representative video of three is shown. DiVX software should be used to open and play this video.
(AVI)

Video S3 **Migration of human monocytes towards SgG2.** Freshly isolated monocytes from blood of healthy donors were plated on glass bottom cover slips coated with Poly-D-lysine and VCAM-1. 5 µM SgG2 was dispensed from a micropipette with constant backpressure. Time-lapse video was recorded at 10 seconds interval with DIC optics at 63× magnification.
(MOV)
