# Supplementary material for: Enhancement of Chemokine Function as an Immunomodulatory Strategy Employed by Human Herpesviruses
Source: PLoS Pathog. 2012 Feb 2;8(2):e1002497. doi: 10.1371/journal.ppat.1002497 (PMC3271085; doi:10.1371/journal.ppat.1002497)
Supplement: Protocol S1 — Generation of recombinant baculoviruses and purification of recombinant proteins. Description of the procedure employed to generate recombinant baculoviruses and purify recombinant proteins. (DOC) [file ppat.1002497.s001.doc]

**Protocol S1: Generation of recombinant baculoviruses and purification of recombinant proteins.**

The honeybee melittin secretion signal peptide present in pMelBac (Invitrogen) was cloned into the pFastBac plasmid (Invitrogen) using *EcoR*V and *BamH*I to excise the melittin secretion signal and *SnaB*1 and *BamH*I to introduce it into pFastBac to produce pFastBacMel. The presence of the melittin signal peptide within the pFastBacMel plasmid was confirmed by sequencing reactions. Truncated forms of gG from HSV-1 or HSV-2 (SgG1 and SgG2, respectively) were amplified by PCR using DNA from HSV-1- or HSV-2-infected cells as templates and primers gG1-3 (*BamH*I) and gG1-2 (*Sph*I) for gG1 and gG2-2 (*BamH*I) and gG2-7 (*Sph*I) for gG2. The amplified DNA products were cloned into pFastBacHTb (Invitrogen). A PCR reaction using oligos pFBHTF (*EcoR*I) and gG1-2 or gG2-7 (*Sph*I) and pFastBacHTb-gG1s or -gG2s as templates was performed to amplify truncated His-tagged-gG1s or -gG2s, respectively, and to clone them into pFastBacMel using *EcoR*I and *Sph*I. All constructs were sequenced to confirm the absence of undesired mutations. Details of the primers will be provided upon request.

The pFastBacMel-HisgG1s or -HisgG2s recombinant plasmids were transformed into competent DH10Bac bacteria where the corresponding recombinant bacmids were generated by a transposition event. Purified bacmids were transfected into Hi-5 insect cells using Cellfectin (Invitrogen) in order to obtain recombinant baculoviruses. The supernatant from the transfected cells was collected 72 h post-transfection and the viruses were amplified in Hi-5 or Sf9 cells. In order to express high amounts of recombinant proteins, Hi-5 cells were infected and the supernatant was collected 72 hours post-infection. Recombinant proteins were purified by affinity chromatography. Purified recombinant proteins were analyzed by SDS/PAGE and Coomassie blue staining or by SDS/PAGE followed by western blotting using an anti-His antibody. SgG1 and SgG2 were also detected with monoclonal anti-gG1 or anti-gG2 antibodies, respectively (LP-10 for gG1 [1]; 4a5a9 for gG2 [2]). BHV-5, EHV-1 and PRV SgGs were expressed, purified and characterized as previously described [3,4].
