# Supplementary material for: Enhancement of Chemokine Function as an Immunomodulatory Strategy Employed by Human Herpesviruses
Source: PLoS Pathog. 2012 Feb 2;8(2):e1002497. doi: 10.1371/journal.ppat.1002497 (PMC3271085; doi:10.1371/journal.ppat.1002497)
Supplement: Protocol S2 — Recombinant chemokines. Relation of recombinant chemokines used in this report. (DOC) [file ppat.1002497.s002.doc]

**Protocol S2: Recombinant chemokines.**

Recombinant chemokines used in the Biacore X biosensor (hCCL1, hCCL2, hCCL3, hCCL3L1, hCCL4, hCCL4L1, hCCL5, hCCL7, hCCL8, hCCL11, hCCL13, hCCL14, hCCL15, hCCL16, hCCL17, hCCL18, hCCL19, hCCL20, hCCL21, hCCL22, hCCL23, hCCL24, hCCL25, hCCL26, hCCL27, hCCL28, hCXCL1, hCXCL2, hCXCL3, hCXCL4, hCXCL5, hCXCL6, hCXCL7, hCXCL8, hCXCL9, hCXCL10, hCXCL11, hCXCL12α, hCXCL12β, hCXCL13, hCXCL14, hCXCL16, hXCL1, hCX3CL1) and the cell migration assays were obtained from PeproTech (London. U.K.), with the exception of hCCL25, hCXCL12α, β and hCXCL13, which were from R&D Systems (Minneapolis, MN). Recombinant [^125^I]-hCCL25, [^125^I]-hCXCL12α and [^125^I]-hCXCL10 were purchased from Amersham.
