# Supplementary material for: Enhancement of Chemokine Function as an Immunomodulatory Strategy Employed by Human Herpesviruses
Source: PLoS Pathog. 2012 Feb 2;8(2):e1002497. doi: 10.1371/journal.ppat.1002497 (PMC3271085; doi:10.1371/journal.ppat.1002497)
Supplement: Protocol S3 — Cross-linking experiments. Explanation of the method used to perform cross-linking. (DOC) [file ppat.1002497.s003.doc]

**Protocol S3: Cross-linking experiments.**

Cross-linking experiments were performed as previously described [5] using [^125^I]-hCCL25, [^125^I]-hCXCL12α, or [^125^I]-hCXCL10 and supernatant from recombinant baculovirus-infected Hi-5 cells, purified recombinant SgGs or Optimem (Gibco) supernatant from mock- or HSV-2-infected cells. As crosslinker, EGS or BS^3^ were used.
