# Supplementary material for: Enhancement of Chemokine Function as an Immunomodulatory Strategy Employed by Human Herpesviruses
Source: PLoS Pathog. 2012 Feb 2;8(2):e1002497. doi: 10.1371/journal.ppat.1002497 (PMC3271085; doi:10.1371/journal.ppat.1002497)
Supplement: Protocol S4 — Cells and viruses. Cells and viruses utilized in this report. (DOC) [file ppat.1002497.s004.doc]

**Protocol S4: Cells and viruses.**

MOLT-4 (human, peripheral blood, leukemia, T cell), MonoMac-1 (human, monocyte-like) were grown in RPMI 1640 (Sigma) containing 10% foetal bovine serum (R-10 medium). BHK-21 and Vero cells were grown in DMEM containing 5% foetal bovine serum. m300-19 cells (mouse B cells) and m300-19-hCXCR5 (stably transfected with hCXCR5) were a gift of Dr. Bernhard Moser (Cardiff University) and were grown in R-10 medium supplemented with β-mercaptoethanol (5 x 10^-5^ M) and sodium pyruvate (1%). m300-19-hCXCR5 were also supplemented with puromycin (1.5 μg/ml). All mammalian cell lines were grown at 37ºC, 5% CO_2_ in a humidified incubator. Sf9 and Hi-5 insect cells were cultured in TC-100 medium (Invitrogen) containing 10% foetal bovine serum. When necessary, adherent Hi-5 insect cells were grown in EX-Cell 405 medium (SAFC Biosciences) to avoid the presence of foetal bovine serum. Conditioned Hi-5 cells were grown as suspension cultures in serum-free Five Express medium (Invitrogen). All insect cells were grown at 28ºC. HSV-1 wild type (wt), strain SC-16, HSV-1ΔgG and HSV-2 wt strain 333 were a gift from Dr Helena Browne (Cambridge, UK). HSV-1ΔgG has been described previously [6].
