# Supplementary material for: Enhancement of Chemokine Function as an Immunomodulatory Strategy Employed by Human Herpesviruses
Source: PLoS Pathog. 2012 Feb 2;8(2):e1002497. doi: 10.1371/journal.ppat.1002497 (PMC3271085; doi:10.1371/journal.ppat.1002497)
Supplement: Protocol S5 — Chemokine binding to infected cells. Description of the method employed to analyze binding of radiolabeled chemokine to infected cells. (DOC) [file ppat.1002497.s005.doc]

**Protocol S5. Chemokine binding to infected cells.**

BHK-21 cells were seeded at high confluency and infected 16-20 hours later with HSV-1 wt or HSV-1ΔgG at a high multiplicity of infection. 14-16 hours post-infection, cells were washed with PBS and incubated with [^125^I]-hCXCL10 during 1 hour. Following the incubation period the cells were scraped, washed three times with PBS, subjected to phthalate oil centrifugation, washed twice with PBS, and cell-bound chemokine was determined using a gamma-counter.
