# Supplementary material for: Enhancement of Chemokine Function as an Immunomodulatory Strategy Employed by Human Herpesviruses
Source: PLoS Pathog. 2012 Feb 2;8(2):e1002497. doi: 10.1371/journal.ppat.1002497 (PMC3271085; doi:10.1371/journal.ppat.1002497)
Supplement: Protocol S6 — Competition of chemokine binding to cells. Explanation of the procedure performed to determine the effect of HSV SgG on radiolabeled chemokine binding to cells. (DOC) [file ppat.1002497.s006.doc]

**Protocol S6: Competition of chemokine binding to cells.**

Competition experiments were carried out incubating 0.5 pmol of [^125^I]-hCCL25 or [^125^I]-hCXCL12α with or without different concentrations of SgGs (or baculovirus supernatants) at 4ºC in binding medium (RPMI 1640 containing 1%FBS and 20mM HEPES pH 7.4) during 1 h at 4ºC. Then 3 x 10^6^ MOLT-4 or MonoMac cells were added to the mixture and incubated for further 2 h at 4ºC with gentle agitation, subjected to phthalate oil centrifugation, washed twice with PBS, and cell-bound chemokine was determined using a gamma-counter.
