# Supplementary material for: Enhancement of Chemokine Function as an Immunomodulatory Strategy Employed by Human Herpesviruses
Source: PLoS Pathog. 2012 Feb 2;8(2):e1002497. doi: 10.1371/journal.ppat.1002497 (PMC3271085; doi:10.1371/journal.ppat.1002497)
Supplement: Text S1 — Includes references used in Protocol S1, Protocol S3, Protocol S4. (DOC) [file ppat.1002497.s007.doc]

**Text S1:**

1. Richman DD, Buckmaster A, Bell S, Hodgman C, Minson AC (1986) Identification of a new glycoprotein of herpes simplex virus type 1 and genetic mapping of the gene that codes for it. J Virol 57: 647-655.

2. Liljeqvist JA, Trybala E, Hoebeke J, Svennerholm B, Bergstrom T (2002) Monoclonal antibodies and human sera directed to the secreted glycoprotein G of herpes simplex virus type 2 recognize type-specific antigenic determinants. J Gen Virol 83: 157-165.

3. Bryant NA, Davis-Poynter N, Vanderplasschen A, Alcami A (2003) Glycoprotein G isoforms from some alphaherpesviruses function as broad-spectrum chemokine binding proteins. EMBO J 22: 833-846.

4. Viejo-Borbolla A, Munoz A, Tabares E, Alcami A (2010) Glycoprotein G from pseudorabies virus binds to chemokines with high affinity and inhibits their function. J Gen Virol 91: 23-31.

5. Alcami A, Symons JA, Collins PD, Williams TJ, Smith GL (1998) Blockade of chemokine activity by a soluble chemokine binding protein from vaccinia virus. J Immunol 160: 624-633.

6. Balan P, Davis-Poynter N, Bell S, Atkinson H, Browne H, et al. (1994) An analysis of the in vitro and in vivo phenotypes of mutants of herpes simplex virus type 1 lacking glycoproteins gG, gE, gI or the putative gJ. J Gen Virol 75 ( Pt 6): 1245-1258.
